# Temperate Forest Floors: Ecosystem Hubs in Transition?

**DOI:** 10.1111/gcb.71033

**Published:** 2026-08-18

**Authors:** Friederike Lang, Jörg Prietzel, Frank Hagedorn, Klaus Kaiser, Stefanie Schulz, Mathias Mayer, Lars Vesterdal, Jürgen Bauhus, Maï Bergmann, Sebastian Bibinger, Martin I. Bidartondo, Jingxuan Olivia Chen, Philipp de Jong, Trung Hieu Doan, Simon Haberstroh, Jonas Hahn, Peter Hartmann, Anis Mahmud Khokon, Martin Kohler, Laura M. Suz, Ina C. Meier, Richard Neumann, Heinke Paulsen, Heike Puhlmann, Helmer Schack‐Kirchner, Stefan Scheu, Lexie Schilling, Patrick Schleppi, Michael Schloter, Kai Schwärzel, Kenton P. Stutz, Gabriela Villalba, Melissa Wannenmacher, Markus Weiler, Nicole Wellbrock, Christiane Werner, Jörg Niederberger

**Affiliations:** ^1^ Chair of Soil Ecology, Institute of Forest Sciences University of Freiburg Freiburg Germany; ^2^ Cluster of Excellence, Future Forests (EXC 3127) University of Freiburg Freiburg im Breisgau Germany; ^3^ Chair of Restoration Ecology, TUM School of Life Sciences Technical University Munich Freising Germany; ^4^ Soil Biogeochemistry, Forest and Soil Ecology Swiss Federal Institute for Forest, Snow and Landscape Research (WSL) Birmensdorf Switzerland; ^5^ Soil Science and Soil Protection Martin Luther University Halle‐Wittenberg Halle (Saale) Germany; ^6^ Research Unit Comparative Microbiome Analysis Helmholtz Center Munich Neuherberg Germany; ^7^ Climate and Biodiversity, Institute of Forest Ecology, Department of Ecosystem Management BOKU University Vienna Austria; ^8^ Department of Geosciences and Natural Resource Management University of Copenhagen Copenhagen Denmark; ^9^ Chair of Silviculture, Institute of Forest Sciences University of Freiburg Freiburg Germany; ^10^ Functional Forest Ecology, Department of Biology University of Hamburg Hamburg Germany; ^11^ Life Sciences Imperial College London London UK; ^12^ Ecosystem Stewardship Royal Botanic Gardens Kew UK; ^13^ Animal Ecology, JFB Institute of Zoology and Anthropology University of Göttingen Göttingen Germany; ^14^ Soil Resources, Department of Environmental Systems Science ETH Zurich Zürich Switzerland; ^15^ Chair of Ecosystem Physiology, Institute for Earth and Environmental Sciences University of Freiburg Freiburg Germany; ^16^ Department of Soils and Environment Forest Research Institute Baden‐Wuerttemberg Freiburg Germany; ^17^ Biosciences Division Oak Ridge National Laboratory Oak Ridge Tennessee USA; ^18^ Center for Bioenergy Innovation Oak Ridge National Laboratory Oak Ridge Tennessee USA; ^19^ Institut Botànic de Barcelona (IBB), CSIC‐CMCNB Barcelona Spain; ^20^ Thünen Institute of Forest Ecosystems Eberswalde Germany; ^21^ Chair of Hydrology, Institute for Earth and Environmental Sciences University of Freiburg Freiburg Germany; ^22^ Centre of Biodiversity and Sustainable Land Use University of Göttingen Göttingen Germany; ^23^ Chair for Environmental Microbiology, Department for Life Sciences Technical University of Munich Freising Germany

## Abstract

The forest floor (FF) plays a key role in carbon, nutrient, and water cycling. It is the biologically most active compartment of forest soils, highly responsive to environmental conditions. Yet, its response to current changes in environmental conditions and forest management is understudied. Temperate forests are among the best studied ecosystems globally, providing the necessary ecological and biogeochemical background information to assess FF changes. Here, we focus on identifying existing knowledge and gaps in our understanding of the functioning of the FF. Interactions between FF biota and abiotic FF components show multifactorial dependencies with environmental conditions and drive FF turnover. Vice versa, the turnover of FF regulates carbon, nutrient, and water cycling. With slow litter decomposition and limited bioturbation, organic matter accumulates, nutrients cycle tightly within the FF, and water passes through this layer partly along preferential pathways. With rapid litter decomposition and intense bioturbation, FF accumulation is little, the mineral soil is the main nexus for plant nutrient uptake and organic matter transformation, and water infiltrates the mineral soil more homogeneously. The interconnectedness with the adjacent ecosystem compartments is a crucial feature of the FF, feeding back to its functioning and making it a central hub of forest processes. The FF morphology reflects these processes and therefore has untapped potential as an indicator of soil and ecosystem health. Under forest change, the FF might lose its functionality, with negative impacts on nutrient provision, water storage, and carbon sequestration. Consequences for forest growth could be strong and even detrimental. Hence, improved knowledge of FF characteristics and their linkages to mineral soils and aboveground ecosystem compartments is crucial for assessing forest resilience to progressing environmental changes.

## Graphical abstract

The forest floor (FF) is a key component of carbon, nutrient, and water cycling and represents the most biologically active part of forest soils. Despite its importance, its response to environmental change and forest management remains insufficiently studied. This review (1) summarizes the ecosystem services provided by the FF, (2) assesses its vulnerability to environmental change, and (3) identifies knowledge gaps regarding its future functioning. Interactions between biotic and abiotic components regulate FF turnover and its role in carbon, nutrient, and water dynamics. Slow litter decomposition and limited bioturbation promote organic matter accumulation, tight nutrient cycling, and preferential water flow, whereas rapid decomposition and intense bioturbation reduce FF accumulation and shift nutrient and water processes to the mineral soil. FFs emerge from the interactive adaptation of organisms to a combination of environmental factors. Thus, multifactorial dependencies and tight interconnectedness with the adjacent ecosystem compartments are crucial to the functioning of FF. Environmental change may have a disconnecting effect, reducing nutrient supply, water storage, and carbon sequestration, with potentially severe consequences for forest growth. Improved understanding of FF characteristics and their links to other ecosystem compartments is therefore essential for assessing forest resilience to progressing changes.

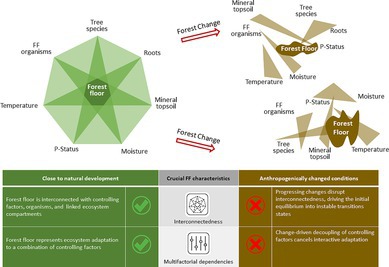

## 1 Knowledge on Forest Floors is Insufficient to Assess Their Response to Forest Change

In the forest floor (FF), organic matter, nutrients and energy of plant, animal, and microbial detritus are transformed, transiently stored, and/or transferred to other ecosystem compartments. This results from complex interactions between plants, organisms, litter, and the underlying mineral soil. The FF provides central ecosystem services by supplying trees with water and nutrients, processing carbon (C), and harboring a wide diversity and abundance of biota, with strong feedbacks to the FF properties and the element cycles within the FF. However, FF organic matter often turns over at higher rates than in the mineral soil (Moreno‐Duborgel et al. 2025; von Fromm et al. 2025), implying that the FFs are highly responsive to the ongoing global “forest change” (Seidl et al. 2014; McDowell et al. 2020), encompassing climate change, nitrogen eutrophication, as well as shifts in tree species composition and overall forest management intensity (Callesen et al. 2003; Vesterdal et al. 2013; Mayer et al. 2020; Mayer, Rusch, et al. 2023; Klein‐Raufhake et al. 2025). Consequently, FF changes can be expected in near future, and there is evidence that FF stocks are already responding to changing environmental conditions (Wellbrock et al. 2017; von Fromm et al. 2025).

**The temperate climate zone as model environment for understanding the causes for and consequences of forest floor changes**. Forest floors (FFs) consist of plant litter in various stages of decomposition overlying the mineral soil. They exist in all climate zones. In this review, we focus on the role of FFs in temperate climates, and their vulnerability to forest change. We identify key characteristics of FFs and related research questions. For the following reasons, we consider the temperate zone to be the ideal model environment for studying FF changes:

***High sensitivity of transition zones***: It is widely assumed that FF thickness is related to climate, with thin FFs atop mineral soils in the warm and moist tropics and thick FFs in cool boreal regions (Olson 1963; Vogt et al. 1986). Temperate regions are characterized by large variations in the thickness of organic layers (Ponge et al. 2011; Hengl et al. 2014), indicating that small changes in environmental conditions can cause distinct changes in FF masses, properties, and functions.

***Profound knowledge on formation and functioning available, but current forest floor changes are understudied***: Although there have been recent advances in understanding of FF formation and classification (Zanella et al. 2022; Wachendorf et al. 2023), they are hardly considered in current global change research. Consequently, recent studies increasingly call for more consideration of FFs (Bernier 2018; von Fromm et al. 2025; Studer et al. 2026).

***Evidence for current changes in temperate forest floors***: Recent studies based on long‐term data series provide evidence that FFs in the temperate zone are changing rapidly (Wellbrock et al. 2017; Prietzel, Hiesch, et al. 2020; von Fromm et al. 2025). In addition, field experiments show that temperate FFs are particularly sensitive to forest disturbance, management, drought, and warming (Davidson and Janssens 2006; Vesterdal et al. 2013, Guidi et al. 2023; Mayer, Rusch, et al. 2023), and thus, may serve as an early‐warning soil indicator (von Fromm et al. 2025).

Consequently, we predominantly covered literature from temperate regions. To highlight general facts and processes independent of climate, we also considered studies conducted under other climatic conditions.

Traditionally, the FF has been considered a key aspect of ecological forest site assessment and survey (Green et al. 1993), especially in boreal and temperate regions. Nevertheless, it has frequently been ignored in forest research (Wan et al. 2023; Neumann et al. 2025). Litter and more decomposed FF layers are removed before soil sampling in many studies (see review by Berthrong et al. 2009). Other studies refer to depth‐related soil analyzes without specifying if FF, mineral soil, or a mixture of both is addressed, which hampers proper evaluation of results (e.g., Lembrechts et al. 2022). One reason for neglecting the FF is its temporal and spatial variability, as well as methodological and analytical challenges (Vogt et al. 1983; Yanai et al. 2003). In result, FFs are poorly represented in forest soil water or biogeochemical models, usually as a simple litter compartment (Peltoniemi et al. 2007; Neumann et al. 2025). Under a changing climate, several FF services have become increasingly critical, yet our knowledge remains limited. Our aim is to compile existing knowledge and identify gaps in our understanding of the vulnerability of FFs to current changes. We addressed this by synthesizing documented evidence of (1) the interplay of individual FF properties—including morphology and taxonomic features—and ecosystem functioning, and (2) the impact of changing environmental conditions on FF characteristics.

## 2 Definitions, Classifications and the Terminology Used in This Review

We refer to “forest floor” as the surface layers of forest soils mainly consisting of litter in various stages of decomposition or transformation (Prescott and Vesterdal 2021) and covering mineral soil horizons or bedrock. There is no internationally standardized terminology for describing the FF. There is not even an agreement as to whether this organic material belongs to the soil or represents an individual ecosystem compartment. Common terms such as organic layer, litter layer, humus layer, humipedon, and forest floor are often used inconsistently (see reviews by Berthrong et al. 2009; Chertov and Nadporoszkaya 2018). A Web of Science search using the terms “litter layer” and “forest” (performed January 2025) revealed that in 30% of the first 50 matches (ranked by decreasing number of citations) the term *litter layer* was used exclusively for freshly fallen, undecomposed litter (IUSS Working Group WRB 2022), while in the other 70% *litter layer* was used as a synonym for the FF, as done in IPCC reports (IPCC 2023). This inaccuracy can easily lead to misinterpretations.

The horizons of FFs are, as mineral soil horizons, strata with a set of characteristic properties and are also termed layers. They are differentiated according to the state of decomposition as indicated by the share of fine humus material (Zanella, Ponge, and Briones 2018; IUSS Working Group WRB 2022; Wachendorf et al. 2023; Table S1): (1) The litter layer (Ol) is hardly decomposed and the origin of the litter can easily be identified, (2) the fermented/fragmented layer (Of; Oi/Oe), a horizon of intermediate state of decomposition, with still identifiable plant tissue, and (3) the humic layer (Oh; Oa), a horizon at an advanced state of decomposition, dominated by organic fine material of macroscopic unidentifiable origin. Due to the high interannual variability, the World Reference Base for Soil Resources (WRB) 4th edition and the US soil taxonomy treat the litter layer as a separate entity rather than a part of the FF (IUSS Working Group WRB 2022; Soil Survey Staff 2022).

The term *humus form* describes the type of the FF, representing its development (Lejoly et al. 2026). Criteria for the assignment of humus forms include the sequence of organic layers (Table S2), specific properties of the underlying uppermost mineral horizon, as well as the different boundaries between the FF and the mineral soil. Seven out of the 11 national soil classification systems reviewed define humus forms and organic horizons (Table S3). We conducted a literature survey (Web of Science), using the topics (browse title, keywords, and abstract) “forest floor” AND (*mull OR *moder OR *mor). The search yielded 92 relevant hits for the publication period 1980 to 2026, while the search for the term “forest floor” without additional filters yielded 9906 hits. The share of publications considering the FF and humus forms decreased strongly within the last decade. Despite current advancements in FF classification (Zanella et al. 2022; Wachendorf et al. 2023), they are hardly applied in current research on global change impacts. Yet, recent studies increasingly call for more consideration of humus forms since providing valuable information on FF formation and associated ecosystem processes (von Fromm et al. 2025; Bernier 2025; Studer et al. 2026).

**FIGURE 1:**
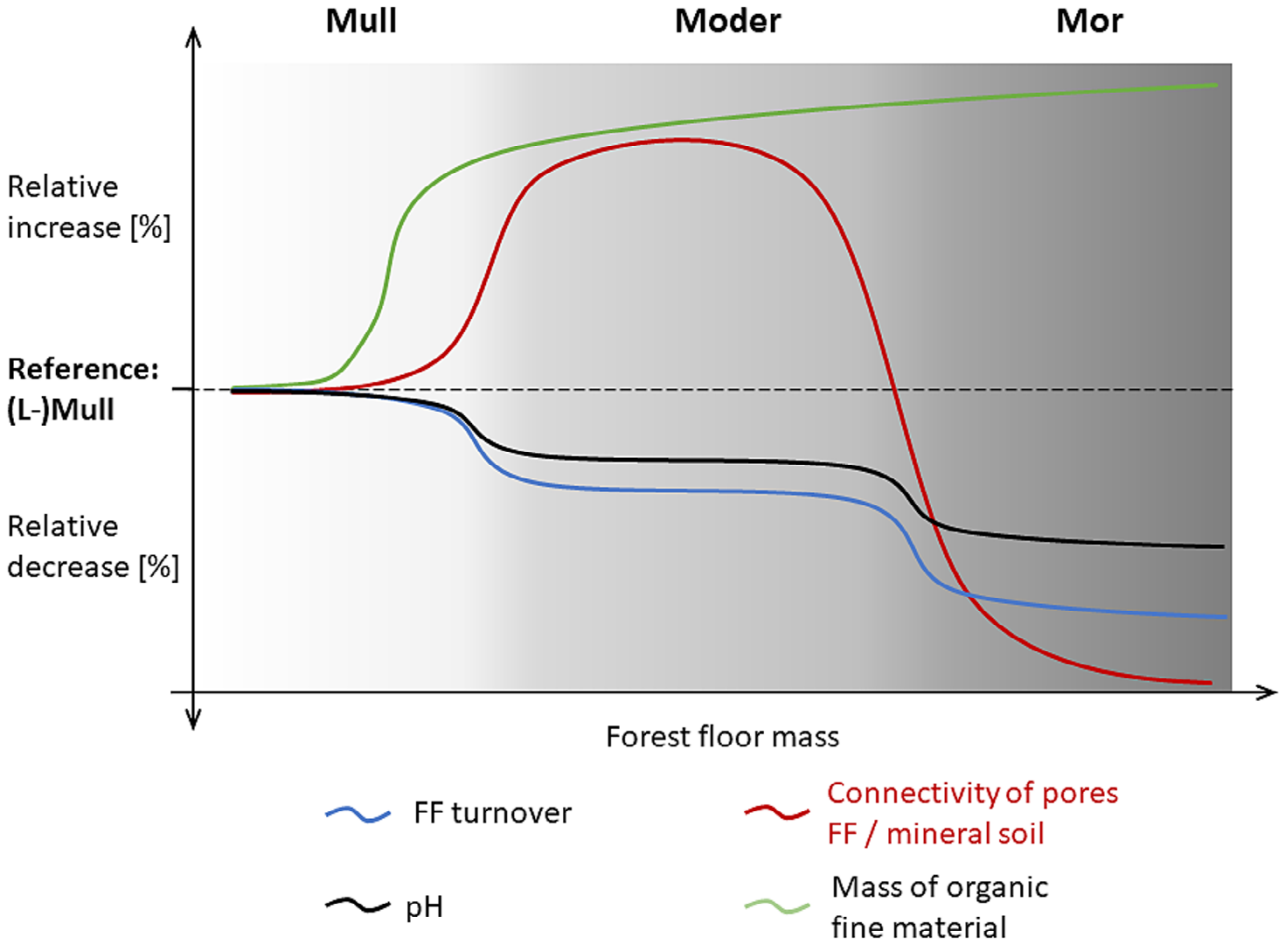
Differences in pH across the major humus forms regulate decomposition and bioturbation, with consequences for forest floor (FF) mass and key properties. The conceptual model assumes constant litter input for all humus forms. Different shades of gray represent the humus forms, without strict differentiation in terms of mass. Proposed changes (specific FF mass minus L‐mull value) are referenced to the L‐mull value.

Mull, moder, and mor are the three major humus forms, representing a gradient of environmental properties and biological processes controlling the rate of decomposition and bioturbation of litter, and thus, the FF turnover (Figure 1) (Ponge 2003; Zanella et al. 2018b; Prescott and Vesterdal 2021; Wachendorf et al. 2023; Zampedri et al. 2023; Lejoly et al. 2026), with mean turnover times of temperate FFs ranging between 1 and 33 years (Vogt et al. 1986). Humus forms have even been assumed to indicate different ecosystem strategies (Bernier 2018). Mull occurs at sites with favorable pH conditions, rich in plant‐available nutrients, highly degradable litter, sufficient precipitation, and temperate to warm climates. It consists of thin organic layers poor in organic fine material due to intense bioturbation, favoring fast below‐ground transfer and decomposition of litter. Some classification systems also distinguish between L‐mull (Of horizons not present; humus form with maximum turnover) and F‐mull (FFs having Ol and Of horizons; Wachendorf et al. 2023). Moder occurs at sites with intermediate conditions and has at least one horizon with a high share of organic fine material (Oh), usually in well‐aggregated form due to less intense incorporation of litter into the mineral soil by bioturbation, but still features high biological activity. Mor typically occurs at acidic sites with a cool and moist climate and low nutrient content of the mineral soil. It consists of thick humus layers that are, in typical form, assumed to be decoupled from the mineral soil and develops where soil fauna plays a minor role for litter transformation and translocation (Lejoly et al. 2026).

The mass of FF or the mass of different FF horizons is not considered in classification. However, it is widely assumed that the FF mass increases from mull over moder to mor, while FF turnover rates decrease (Klein‐Raufhake et al. 2025; Figure 1). With different humus forms, FF characteristics change substantially as represented conceptually in Figures 1 and 3a–e by points of inflection at the transition between different humus forms. For example, the presence of an Oh horizon rich in organic fine material in moder aligns the pore size distribution at the lower boundary of the FF more closely with that of the underlying mineral soil than in mull‐type FFs (Figure 1). Consequently, pore connectivity strongly increases at the transition from mull to moder (Paulsen and Weiler 2026). If FF mass accumulates further and living conditions of fauna in the mineral topsoil become less favorable, mor‐type FFs form. Fine roots and fungal hyphae preferentially grow at the interface between FF and mineral soil and disrupt pores (Zanella et al. 2018a; Wachendorf et al. 2023). Thus, connectivity shows a steep decrease from moder to mor.

## 3 The Relevance of Forest Floors for Ecosystem Functioning

**FIGURE 2:**
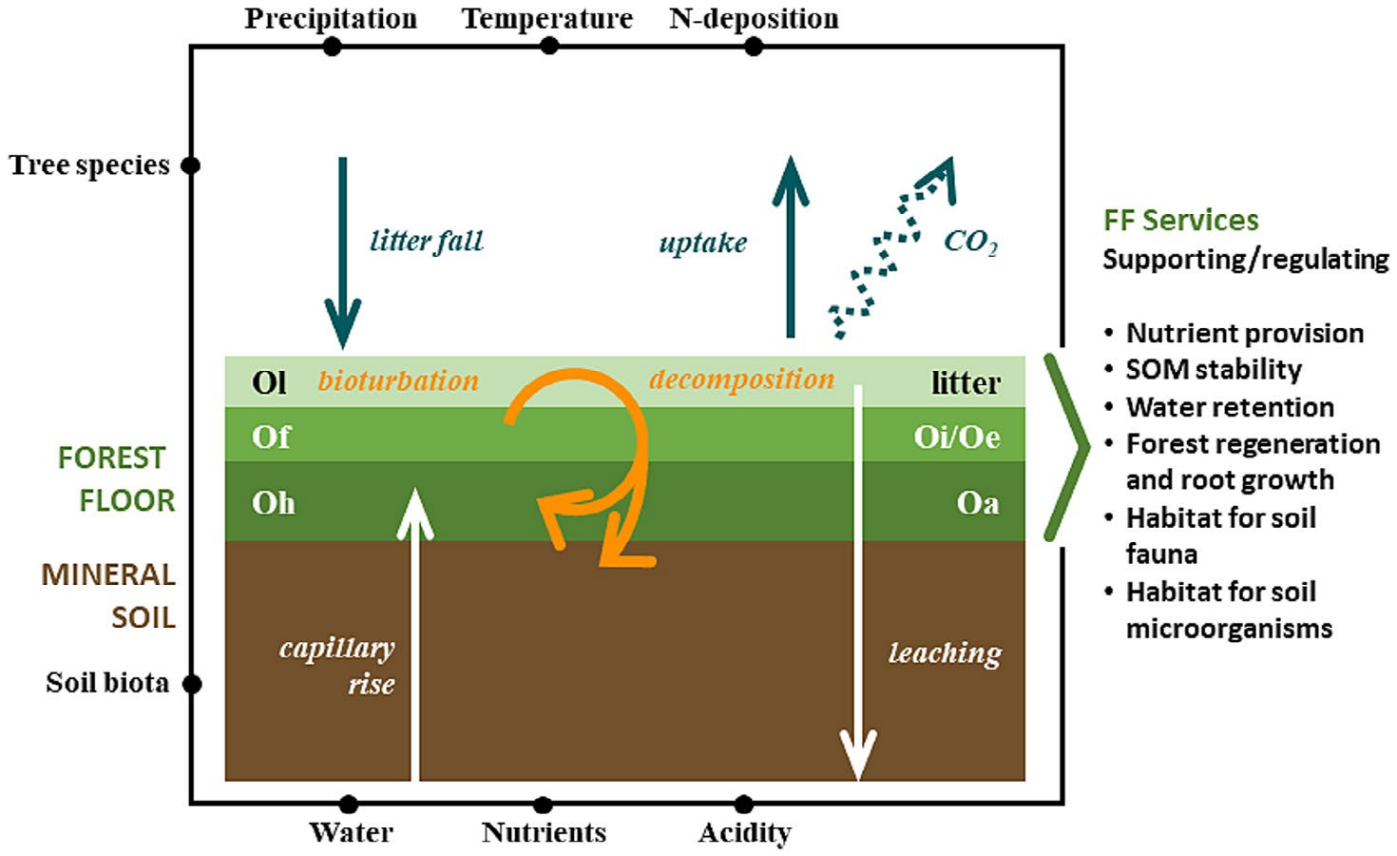
Factors controlling forest floor (FF) characteristics and related target ecosystem services, Ol, Of, Oh: Organic horizons according to the German classification system (Wachendorf et al. 2023), litter, Oi/Oe, Oa: Organic horizons according to WRB (IUSS Working Group WRB 2022). The fulfillment of the services listed in the figure is outlined in the following sections.

Controlling factors of FF dynamics and properties include climate, parent material, vegetation, soil organisms, and the processes they induce. Turnover of FF, mirrored by its mass, thickness, or humus form, depends on litter and root input, living conditions for decomposers, and the organisms involved in bioturbation (Figure 2). The FF turnover increases with temperature, moisture (if oxygen is not limiting), nutrient availability, and litter decomposability (Grigal and Vance 2000), resulting in decreasing FF thickness. The relevance of the given controls is widely accepted (Grigal and Vance 2000; Moreno‐Duborgel et al. 2025). However, there are many unknowns regarding the quantitative importance of these factors and their interactions (Rahman and Tsukamoto 2015). As a major reservoir of organic matter and nutrients, the FF plays a vital role in regulating ecosystem processes and associated ecosystem services (Gosz et al. 1976; Figure 2). Nutrient, C, and water cycling in forests can completely change with changing FF turnover and with changing FF controls (Bernier 2018).

### 3.1 The Role of the Forest Floor for the Mineral Nutrition of Trees Within Ecosystems

**FIGURE 3:**
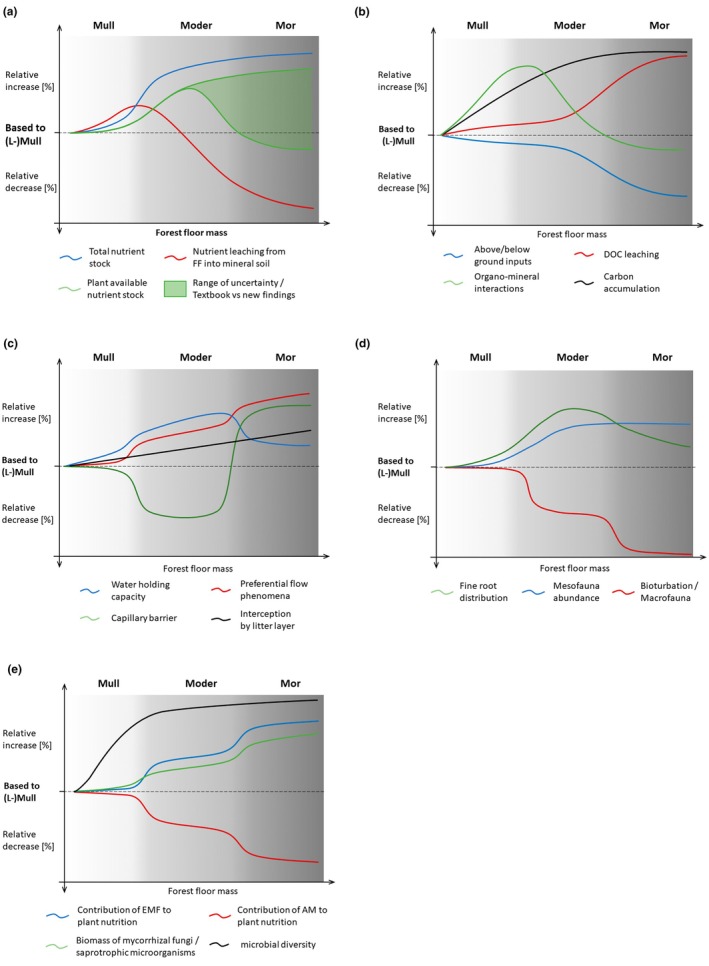
Various characteristics of FFs as depending on FF mass and humus form – all values are referenced to that of L‐mull. (a) Total nutrient stocks, plant availability, and nutrient leaching from forest floor (FF) into the mineral soil depending on FF mass and humus form. (b) Share of organic matter involved in organo‐mineral interactions, carbon accumulation, ratio between above and below‐ground input of organic matter into forest floor (FF), and transfer of DOC from FF into the mineral soil as depending on FF mass and humus form. (c) Interception by the litter layer, preferential flow, water holding capacity, and capillary barrier, indicating the relevance of the forest floor (FF) for water dynamics in forest soils as depending on FF mass and humus form. (d) The relevance of the forest floor (FF) for tree roots and the soil fauna as depending on FF mass and humus form. (e) The relevance of the forest floor (FF) for mycorrhizal fungi and microbial diversity, contribution of mycorrhiza to plant nutrition and the ratio of mycorrhizal fungi biomass to saprotrophic microorganisms as depending on FF mass and humus form.

The FF first received attention in ecology because of the observed negative relation between FF thickness and forest productivity (Babel 1971). Thin, mull‐type FFs have been assumed to indicate high fertility linked to high litter quality and fast release of nutrients, while thick, mor‐type FFs have been assumed to indicate low biological activity and nutrient availability (Müller 1887; Olson 1963; Hartmann 1965; Green et al. 1993; Bardgett 2005). Yet, it must be considered that thick FFs are often the consequence rather than the cause of low fertility. Thick FFs are important for forest nutrition at sites with nutrient‐poor mineral soil, which is particularly well‐established regarding phosphorus (P) (Paré and Bernier 1989; Jonard et al. 2009; Lang et al. 2017; Prietzel et al. 2022). Studies quantifying the contribution of FFs to overall nutrient uptake and cycling in forests are scarce. Lang et al. (2017) and Prietzel et al. (2022) presented rough estimates for beech forests based on the share of overall fine root biomass present in the FF. Depending on parent material and P availability at the study sites, a minimum of 20% (P‐rich site) to 92% (P‐poor site) of the total P taken up from down to 1 m mineral soil depth was provided by the FF and the upper 5 cm of the mineral soil. Based on isotopic tracing (^26^Mg), van der Heijden et al. (2015) determined that 43% of the magnesium (Mg) take up in a beech forest on acid, nutrient‐poor soil was acquired from the FF. The role of the FF in forest nutrition depends on nutrient stocks, nutrient availability, and rooting intensity, which all change with FF mass (Figure 3a,d) and influence the contribution of FFs to overall tree nutrient uptake.

Leaf litter has long been assumed to be the most important source of FF nutrients (Ukonmaanaho et al. 2008), though root and mycorrhizal necromass are increasingly recognized (Phillips et al. 2013). Mineral particles incorporated into the FF from underlying soil horizons by bioturbation or formed in situ may also contribute to FF nutrient dynamics (Prietzel, Hiesch, et al. 2020; Prietzel et al. 2023). Furthermore, nutrients can be transported into the FF by capillary rise from the mineral soil (Figure 2).

With increasing FF mass, the stock of nutrients tends to increase (Figure 3), but also the proportion of highly decomposed material increases. Therefore, the relationship between the mass and nutrient stocks in FFs depends on litter decomposition stoichiometry and on the fate of mineralized nutrients. Nutrients can be mineralized faster, as fast, or slower than C (Berg et al. 2017; Dincher et al. 2020). Depending on the situation, the increase in nutrient stocks can be slower, same, or faster than the increase in mass of organic matter. Literature reviews on the release of nutrients during litter decomposition indicate element‐specific relations. Synthesizing results of 68 litter decomposition studies mainly of boreal and temperate pine and spruce forests, Berg et al. (2017) observed that calcium (Ca) and potassium (K) concentrations of decomposing litter decreased with time (after a first concentration increase for Ca). Increasing concentrations of manganese (Mn), nitrogen (N), phosphorus (P), and sulfur (S) have been observed at later decomposition stages (Berg et al. 2015; Gunina and Kuzyakow 2022; Covington 1981; Vesterdal et al. 1995). Although often neglected, the ecological relevance of FF internal nutrient cycling has already been stressed in a review by Currie (1999). High relevance of FF internal nutrient cycling has been confirmed by more recent studies on N (Högberg et al. 2020) and P (Hauenstein et al. 2018; Spohn et al. 2018; Brödlin et al. 2019). The FF internal nutrient cycling strongly depends on tree species, their rooting patterns, and associated microorganisms (see detailed below; Smith and Read 2008; Pena et al. 2010; Khokon et al. 2023), and is linked to decreasing nutrient leaching into the mineral soil (Figure 3). Accordingly, Vogt et al. (1986) observed significantly higher mean residence times for N than for C in FFs of coniferous forests than of other ecosystems. This might indicate internal N (re)cycling within the FF, complementing nutrient uptake by plants.

### 3.2 The Role of the Forest Floor in Soil Carbon Dynamics

Carbon stocks in FFs of European forest soils range from 1 Mg C ha^−1^ in thin mull‐type FFs to around 70 Mg C ha^−1^ in thick mor‐type FFs. On average, the FF accounts for 20% of the soil C stock down to 1 m based on a survey of 4914 plots among 22 European countries (De Vos et al. 2015), aligning with a previous survey of European forest soils (Baritz et al. 2010). The humus form explained most of the variability in FF C stocks (De Vos et al. 2015) (Figure 3b). The FF consists of a continuum of compounds ranging from minimally decomposed litter to highly transformed organic matter in Oh horizons. Plant litter is predominant throughout the FF (Cools et al. 2014; Berg and McClaugherty 2020), but with increasing FF depth, the share of plant‐derived tissues (e.g., foliage, wood, bark) and compounds therein (e.g., lignin, cellulose, hemicellulose) decreases relative to microbial metabolites and necromass (Buckeridge et al. 2020; Prescott and Vesterdal 2021).

The Of and Oh horizons additionally receive substantial inputs of root residues as they can harbor considerable root biomass (Guidi et al. 2023). In Norway spruce FFs, the biomass of roots increased strongly from mull‐ to mor‐type FFs, driven both by soil fertility and thinning intensity gradients (Vesterdal et al. 1995; see also Figure 3d). In boreal forests, Fahey and Hughes (1994) and Clemmensen et al. (2013) showed that 50%–70% of the FF‐C stock can be derived from roots and root‐associated microorganisms. Accordingly, the relative contribution of above‐ground C input to the FF will decrease with increasing FF mass (Figure 3b). Although often overlooked, Of and Oh horizons can contain considerable amounts of mineral‐associated organic C, as shown by density fractionation (Villalba‐Ayala et al. 2025). The proportion of mineral‐associated C increases with depth and the degree of decomposition, unless faunal bioturbation is impaired (Figure 3b). As in mineral horizons, mineral association may contribute to C stabilization within the FF (Prietzel, Hiesch, et al. 2020).

The FF is a key reactor for soil C cycling, where litterfall and root‐associated C inputs are either mineralized to CO_2_, transferred into the mineral soil either by leaching as DOC or by soil fauna via bioturbation as POC, or stabilized against further decomposition (Fahey et al. 2013; Hagedorn et al. 2019; Kohl et al. 2021; Figure 3b,d). The relative and quantitative importance of these processes is still uncertain and highly context‐specific (Kammer et al. 2012; Stutz et al. 2019; Desie et al. 2020; Wang et al. 2021). Large‐scale assessments using litterbags revealed that climate variables (mean annual temperature and precipitation) and litter quality, particularly lignin content, C/N ratio, and Mn content, are key factors controlling litter mass loss (Berg and McClaugherty 2020). Much less is known about C lost and transformed during decomposition (Rahman and Tsukamoto 2015). A one‐year study of a mull‐type FF, using ^13^C‐labeled beech litter, revealed that 23%–31% of the added litter‐C remained in the FF, 29%–34% was mineralized, 4%–5% leached as DOC, and circa 30% translocated into the mineral soil via bioturbation (Kammer et al. 2012). For moder‐ and mor‐type FFs, leaching of DOC is of much greater and translocation of C via bioturbation of lower relevance (Qualls 2016). Prescott and Vesterdal (2021) stressed that transformation of litter may cause accumulation of *de novo* materials, such as microbial necromass and faunal detritus, that persist within the FF.

Radiocarbon‐based assessments of FFs indicated that turnover times range from less than 5 years in mull‐type FFs of temperate forests to 150 years in thick mor‐type FFs at alpine tree lines, with much longer turnover times in coniferous than in broadleaved forests with lower FF mass (Moreno‐Duborgel et al. 2025). Nutrient status may matter; under conditions of low nutrient availability, the necromass of ectomycorrhizal fungi may represent a significant and stable C pool in Oh horizons (Clemmensen et al. 2013), while additional mechanisms of stabilization might be in play at high N availability (Högberg et al. 2020; Yeung et al. 2025). Thus, the stability of FF organic matter under current and future conditions remains uncertain and hardly predictable.

### 3.3 The Role of the Forest Floor for Water and Energy Cycles

The FF serves as a critical interface in forest ecosystems, mediating the storage and transfer of water and energy between the atmosphere and mineral soils. Forest floor material is characterized by notably low bulk density (typically < 0.2 g cm^−3^) and high porosity (> 85% v/v; Laurén and Mannerkoski 2001; Greiffenhagen et al. 2006), markedly distinct from mineral soils. Despite the FF's exceptional physical properties, dedicated knowledge of its water retention and transport characteristics remains limited (Keith et al. 2010; Paulsen and Weiler 2025). Water retention in and the related water holding capacity of FFs is a subject of study across various disciplines, often with differing focuses and terminology. From a soil science perspective, research centers on water holding capacity and mid‐term storage within FF pores, while ecohydrology emphasizes the short‐term interception of water on litter surfaces (Zhao et al. 2022). The water holding capacity of FFs is governed primarily by FF mass and humus form (Figure 3c), which are linked to the state of decomposition and the presence of organic fine material (Ilek et al. 2017). Tree species composition also plays a role, probably by effects on FF properties (Ilek et al. 2015, 2017, 2024; Raaflaub and Valeo 2009). Systematic analyzes of the relationship between humus forms, FF mass, and water holding capacity are lacking. Nevertheless, the general trend (Figure 3c) suggests that with progressing decomposition, fine pore volume increases, and water retention rises from mull to moder humus forms (Paulsen and Weiler 2026). In contrast, mor‐type FFs, with less zoogenic transformation, may feature larger pores due to reduced aggregation of fine particles (Paulsen and Weiler 2025). These differences underscore the need for studying FF structure and composition in relation to hydrological function.

Interception of water by the FF (Figure 3c), in particular on the fresh litter layer (Ol), increases with thickness (Molina et al. 2019), respectively its mass (Zhao et al. 2019; Paulsen and Weiler 2025). Subsequent evaporation empties the temporal interception storage rapidly. Interception and subsequent evaporation can affect the forest microclimate by increasing humidity, reducing air temperature, and lowering vapor pressure deficit (Floriancic et al. 2022). Interception storage decreases with slope, as steeper locations promote runoff from leaf surfaces (Zhao et al. 2022), termed biomat flow (Bachmair and Weiler 2011).

The FF forms a vital link between atmosphere and mineral soil, mediating fluxes of water, gas, and heat. Connectivity between FF and mineral soil is shaped by the FF's architecture, including thickness, structure, and wettability (Fekete et al. 2016). These properties, together with the structure of the upper mineral soil, determine how precipitation is partitioned between surface evaporation, subsurface flow, and infiltration, ultimately contributing to the plant‐available water pool of the mineral soil. Water transport through the FF is bidirectional. High root density, atmospheric exposure, and transient vapor pressure deficits can establish strong water‐potential gradients, driving upward water fluxes from mineral soil into the FF. Schaap et al. (1997) found for Douglas fir FF that about 25% of annual FF water loss via evapotranspiration was replenished by capillary rise. Conversely, the FF can hinder downward water fluxes due to water repellency, especially under dry conditions. Water repellency in FFs is observed at higher residual water contents than in mineral soils (Greiffenhagen et al. 2006; Wessolek et al. 2008) and is more pronounced in thicker FFs during dry conditions (Gimbel et al. 2016). The repellency increases surface run‐off in sloping terrain (Neris et al. 2013), alters infiltration dynamics (Orfánus et al. 2021; Gimbel et al. 2016), and can cause preferential flow along wettable microsites (Figure 3c), potentially directing rainwater toward roots (Wessolek et al. 2008).

Another control on hydrological functioning is the difference in pore size distribution between FF and underlying mineral soil, which can create a capillary barrier (Figure 3c). This effect is most pronounced in humus forms with an abrupt change in pore size at the FF–mineral soil interface and may promote shallow subsurface flow (interflow). The FF–mineral soil interface also limits upward water transport, thereby acting as a protective shield that reduces evaporation (Paulsen and Weiler 2026). The shielding effect of the FF has been demonstrated by Floriancic et al. (2022), who found that removing the Ol layer in a temperate mixed forest increased mineral soil evaporation, indicating that FF's protective role can outweigh water loss caused by its interception effect.

The FF also plays a significant role in the soil thermal regime. It (1) absorbs net radiation at the atmospheric boundary, (2) facilitates heat transfer into the mineral soil, and (3) enables lateral heat exchange. Several studies (Vose and Swank 1991; Kang et al. 2000; Paul et al. 2004) have highlighted the FF's importance in moderating soil temperatures beneath different forest canopies. Both the FF and tree canopy layers serve to buffer extreme air temperatures (De Frenne et al. 2019), though the underlying mechanisms remain uncertain. Thermal conductivity in FFs is closely linked to pore size distribution and water content. In mineral soils, solid particles have higher thermal conductivity (1.8–8.8 W m^−1^ K^−1^) than water (0.6 W m^−1^ K^−1^) or air (0.025 W m^−1^ K^−1^), but for organic FF material, thermal conductivity falls between that of water and air (Heiskanen 2000). Larger water‐filled pores increase thermal conductivity, while air‐filled pores decrease it. Removal of FF increases mineral soil temperatures and daily temperature amplitudes (DeLaurentis 2024). The geometry and connectivity of pores, as well as the degree of water saturation, are key determinants of FF thermal conductivity (O'Donnell et al. 2009; Ebel et al. 2019), impacting both direct heat conduction and the movement of energy transporting media.

Despite advances in understanding FF structure and function, clear evidence is still lacking regarding the specific FF properties that control hydrological and thermal processes. Effects of tree species as well as seasonal and spatial variations in FF properties also remain unresolved.

### 3.4 Forest Floors as Habitat for Tree Roots and Seedlings

Forest floors can harbor a great proportion of tree fine roots. For example, total fine root biomass of European beech in the FF increases with elevation and FF thickness (Figure 3d), which was not evident in the mineral soil (Leuschner et al. 2022). In particular, at nutrient‐poor sites, thick FFs provide a suitable environment for plant roots. Here, the risk of low oxygen levels is low since close to the surface, the water holding capacity of the FF is high (Schenk and Jackson 2002; Gao et al. 2021), and readily available nutrients due to litter and organic matter decomposition promote fine root growth (Sayer et al. 2006). Fine roots in the FF show higher absorptive capacity for nutrients than roots in the mineral soil (Bauhus and Messier 1999; Gao et al. 2021), highlighting their primary role in nutrient acquisition, typically in close association with mycorrhizal fungi (see below). However, organically bound nutrients present in the FF are mostly not directly plant‐available and require organic matter decomposition. Providing readily degradable photosynthates in the form of root exudates to mine for nutrients, plants can accelerate the decomposition by the priming effect (Rohrbacher and St‐Arnaud 2016). For example, N cycling can be accelerated by root exudation (Meier et al. 2017). Especially the presence of organic N—as found in the FF—is thought to increase the amount of root exudation (Tückmantel et al. 2017; Vives‐Peris et al. 2020). Such processes might lead to different root exudation rates and root exudation compositions in the FF and the mineral soil (Wannenmacher et al. 2025). The relevance of FFs for roots strongly varies with FF mass, humus form (Figure 3d), mineral soil, and other site conditions as well as with tree species composition and stand conditions (see above, Vesterdal et al. 1995). Recent work indicates that rooting density might be highest in moder‐type FFs, but decreases under acid conditions of mor‐type FFs (Bernier 2025). Yet, systematic analyzes of the relationship between humus forms and rooting density are still lacking.

Roots can strongly contribute to the accumulation or loss of organic C in the surrounding soil (Poirier et al. 2018), either by producing recalcitrant litter or by enhancing stabilization of organic matter based on microbial processing (Sokol and Bardford 2019). However, allocation of high proportions of fine roots to the FF increases the sensitivity of plants to prolonged drought. This is indicated by the exponential decrease in the relative importance of FF as fine‐root habitat in nutrient‐poor forest stands with increasing risk of summer drought (Meier and Leuschner 2008).

FFs play a controversial role for tree regeneration. The early establishment of seedlings consists of different stages that are influenced by the FF serving as seedbed and subsequently as growth substrate. Information on how FF properties affect seedling establishment, both in direction and magnitude, is often ambiguous and context‐ and tree species‐specific (Madsen 1995; Sayer 2006; Major et al. 2013). Forest floors feed positively back to regeneration by their water retention (Tiebel et al. 2023), habitat function for mycorrhizal fungi and their spore banks (Sayer 2006), and shelter against granivores, such as birds and rodents (Myster and Pickett 1993; Hulme and Borelli 1999). On the other hand, thick FFs may act as a mechanical barrier that reduces light transmittance and prevents seedling roots from reaching the mineral soil (Sayer 2006; Baskin and Baskin 2014). Phytotoxins, present in the litter of certain plant species (e.g., *Picea abies* ), can hinder germination and early seedling growth of some woody and herbaceous species (Koorem et al. 2011; Zeidler 2023). Responses to FF conditions vary with seedling ontogeny, making it difficult to determine favorable conditions for establishment success. In a study of 15 North American tree species, increased emergence was found in the absence of litter, but litter presence prolonged post‐emergence survival under drought conditions (Fisichelli et al. 2015). Additionally, the FF properties interact with abiotic (e.g., temperature and precipitation) and biotic factors (e.g., plant competition and fungal pathogens). Thick and wet FF harbors fungal pathogens (Butin 2011) and molluscs, which contribute to tree regeneration failure (Ibáñez et al. 2007; Zimmermann 2009; Fisichelli et al. 2015). Increased survival of moisture‐sensitive species was observed in scarified seedbeds compared to undisturbed FF conditions under drought (Clark and D'Amato 2022). This could be attributed to the more stable water holding capacity of mineral soil relative to hydrophobic dry FF. Regarding plant nutrition, in P‐poor beech forests, FF may be an important source of P for seedlings and mature trees (Lang et al. 2017; Hauenstein et al. 2018). Decreasing FF thickness may intensify the competition for P exerted by dominant trees on seedlings (Coomes and Grubb 1998, 2000).

Given the complex ways by which FFs can influence tree regeneration, our knowledge of how seedling establishment responds to FF conditions is still incomplete. Additional experimental research is essential to develop a mechanistic framework of how FF properties drive vegetation shifts under climate changes. Also, most research on plant establishment has focused on the presence or absence of the litter layer, rather than considering the full range of FF forms and properties (Fisichelli et al. 2015; Wang et al. 2022; Tiebel et al. 2023).

### 3.5 Forest Floors as Habitat for Soil Fauna

Animals completing their full life cycle in soil include macrofauna (e.g., earthworms, millipedes, isopods), mesofauna (e.g., springtails, oribatid mites, enchytraeids), and microfauna (e.g., protists and nematodes) (Swift et al. 1979; Nielsen 2019). Early studies already linked FF formation to the activity of soil animals, particularly arthropods and earthworms (Müller 1887). Soil animal communities are vertically structured, with many species predominantly inhabiting the FF (Decaëns et al. 2006; Berg and Bengtsson 2007). While aboveground litter provides major food resources, root‐derived C also contributes substantially to soil food webs (Zieger et al. 2017; Zhou et al. 2023).

FF horizons provide resources for a broad range of feeding guilds, from primary decomposers consuming plant litter to microbial feeders, predators, and scavengers (Scheu and Falca 2000; Schneider et al. 2004; Chahartaghi et al. 2005; Chen et al. 2025). Microarthropods often dominate FF communities (Mitchell 1978; Arribas et al. 2021) and typically prefer intermediate stages of litter decay (Fujii and Takeda 2017; Marian et al. 2018). Since low‐quality litter is often difficult to consume directly, many detritivores depend on prior microbial processing (Maraun and Scheu 1996; Frouz 2018). The Ol horizon provides habitat for epigeic macrofauna and larger microarthropods, whereas Of and Oh horizons are heavily colonized by microbial feeders and secondary decomposers (Huhta and Hänninen 2001; Zanella et al. 2018a; Figure 3d). Mineral soil is mainly inhabited by small euedaphic microarthropods and burrowing macrofauna (Nielsen 2019). The FF buffers environmental fluctuations and provides a relatively stable microclimate for soil fauna (Walsh and Voigt 1977; Sayer 2006). Organic horizons also offer shelter and breeding sites (Bal 1970; van Straalen 2023). Animal distribution within the soil profile is closely linked to body size. Protists and microfauna occupy water films, mesofauna depend on existing pore networks, whereas macrofauna actively modify their habitat through burrowing and bioturbation (Lavelle and Spain 2002; Erktan et al. 2020).

Soil fauna support important ecosystem functions. They contribute directly to litter decomposition through feeding and fragmentation (Frouz 2018; Joly et al. 2020). Detritivores such as earthworms, millipedes, isopods, springtails, and oribatid mites transform litter into faecal pellets containing finely fragmented organic material (Nielsen 2019; Potapov et al. 2022). Soil animals also indirectly influence decomposition by modifying microbial communities and environmental conditions (Scheu et al. 2005; Frouz 2018). Faecal pellet formation can accelerate decomposition, while grazing on saprotrophic fungi may enhance nutrient mobilization and litter breakdown (Seastedt 1984; Scheu et al. 2005; A'Bear et al. 2014). Across ecosystems, soil fauna increases litter decomposition by an average of 27%, although effects vary with biome and litter quality (Garcia‐Palacios et al. 2013). Soil animals are also key drivers of nutrient cycling. By consuming microbial and fungal biomass, and altering microbial community composition, they influence nutrient availability (Coleman 1985; Bardgett 2005; Scheu et al. 2005). Bacterivorous nematodes release substantial amounts of mineral N, thereby promoting plant growth (Griffiths 1994; Ferris et al. 1998; Laakso et al. 2000). Likewise, earthworms enhance nutrient mineralization and plant productivity (Scheu 2003; Van Groenigen et al. 2014; Fonte et al. 2023).

The composition of soil faunal communities strongly influences humus form development. In mull‐type FFs, macrofauna biomass is high, particularly that of anecic and endogeic earthworms (Jabiol et al. 1995; Parkinson et al. 2004). These organisms redistribute organic matter by bioturbation and incorporate litter into mineral soil (Bouché 1975; Ferlian et al. 2020). In moder humus forms, lower pH and poorer litter quality reduce earthworm abundance and bioturbation intensity, leading to thicker organic horizons (Bouché 1975; Frouz 2018; Edwards and Arancon 2022; Figure 3d). In mor humus forms, macrofaunal activity is minimal and bioturbation largely absent (Bardgett 2005; Frouz 2018). Bioturbation also affects mesofauna communities. In mull FFs, intensive earthworm activity reduces the abundance and diversity of springtails and oribatid mites (Eisenhauer 2010; Ferlian et al. 2018; Jochum et al. 2021). This likely contributes to the increase in mesofauna abundance observed from mull to moder humus forms (Schaefer and Schauermann 1990; Maraun and Scheu 2000; Frouz 2018). The transition from macrofauna‐ to mesofauna‐dominated systems along the mull–moder gradient is closely linked to soil pH. Macrofauna are generally more sensitive to acidity than mesofauna. In millipedes and isopods, this is partly related to reduced availability of Ca needed for cuticle hardening (Hopkin and Read 1992; Zimmer 2007), while earthworms are additionally affected by Al toxicity (Phillips and Bolger 1998; Edwards and Arancon 2022). Consequently, endogeic and anecic earthworms are largely absent from acidic soils, favoring moder development. In northern North America, where native earthworms are scarce, moder‐type FFs also occur on high‐pH soils. Invasion by European earthworms has transformed the FF of many of these systems into mull‐type forms, providing valuable insights into associated shifts in faunal communities, food webs, and ecosystem functions (Alban and Berry 1994; Bohlen et al. 2004; Ferlian et al. 2018).

Overall, the role of macrofauna in litter decomposition and organic matter translocation has received much greater attention than that of mesofauna. Studies investigating the combined effects of macro‐ and mesofauna on FF structure, microbial communities, and humus form development remain scarce.

### 3.6 Forest Floors as Habitat for Microorganisms

The large amount of decomposable organic material in the FF (Lang et al. 2017) supports mineralizing bacteria, mycorrhizal and saprotrophic fungi, and other microeukaryotes. Community composition of microbiota, their diversity and functionality strongly depend on FF type and tree species (Lindahl et al. 2007; Solly et al. 2017; Bonanomi et al. 2019). The FF often represents the zone with the highest microbial activity along the soil profile (Pollierer et al. 2015; Lladó et al. 2017), declining with progressing litter degradation (Baldrian et al. 2013; Žifčáková et al. 2016; Preusser et al. 2019; Buresova et al. 2021) and from mull over moder to mor humus forms (Hellwig et al. 2019).

FF substrate quality changes strongly as litter decomposition progresses, that is with time and along the FF profile, indicating temporal and spatial fluctuation of the microbiome (Žifčáková et al. 2016). In line with the changes in substrate quality, soil respiration and microbial biomass decrease from Ol to Of and Oh layers (Kanerva and Smolander 2007). Typically, the initial stages of litter decomposition are dominated by saprotrophic fungi, such as basidiomycete and ascomycete microfungi (Osono 2007; Eichlerová et al. 2015; Khokon et al. 2021), as well as *Proteobacteria* and *Bacteriodetes*, making up 60% and 30% of the active community in a Norway spruce ( *Picea abies* ) dominated temperate forest, respectively (Žifčáková et al. 2016). Nitrogen‐mobilizing mycorrhizal fungi are more dominant in the Of and Oh layers (Lindahl et al. 2007). In particular, bacteria respond significantly to changes in litter C:N ratio, while fungi are more driven by tree species composition (Urbanová et al. 2015). With FF mass, the heterogeneity of properties along the depth profile is increasing. Thus, the microbial diversity of the FF likely increases with FF mass (Figure 3e).

Despite huge differences in substrate quality (micro‐ and macronutrients) and in C:N:P ratios of different FF layers (Takahashi 2021), the concept of microbial homeostasis (Cleveland and Liptzin 2007) is still a valid concept and a driver of bacterial functional community composition as indicated by similar microbial C:N:P ratios in FF and mineral soil (Zederer et al. 2017). This poses the question at which level of organization microbial regulation in FF takes place: at the level of microbial biomass, at the level of microbial communities, or at the level of gene expression of single microorganisms.

In addition to the FF material itself, living fine roots in the FF provide an additional microhabitat and substrate within Of and Oh layers (Kanerva and Smolander 2007). The rhizosphere serves as a sink as well as a source for nutrients, mainly during the growth period. On the one hand, trees rely on nutrients provided by microbial processes in the soil and mycorrhizal transfers, and directly compete for those, especially N and P. On the other hand, trees provide easily available C sources for microbial nutrition, bypassing the need for the degradation of complex C sources provided by the litter. However, nutrient acquisition strategies differ among tree species. Spruce is known to release nitrification inhibitors, and thus, directly alters microbial N turnover and nutrient stoichiometry (Stempfhuber et al. 2017). Studies of mineral forest soils demonstrated the importance of ammonia‐oxidizing archaea in acidic spruce stands to maintain this important ecosystem function (Stempfhuber et al. 2014). However, it is not clear if this also holds true for the FF. The large amount of fungal biomass in FFs (Högberg and Högberg 2002) makes the hyphosphere another relevant microhabitat, particularly for bacteria, which might use it as a nutrient source, but also as a fungal highway to bypass nutrient‐poor microsites (Meier et al. 2015; Simon et al. 2015; Bielčik et al. 2019). Bacteria were shown to be invaders as well as degraders of fungal cell walls, with the dynamics of decomposition processes strongly depending on the chitin and melanin contents of hyphae.

Most tree species are associated with either arbuscular mycorrhizal fungi (AMF) or ectomycorrhizal fungi (EMF). Trees associated with AMF are typically linked to thinner FFs and faster nutrient mineralization, whereas trees associated with EMF tend to occur with thicker FFs and slower organic matter decomposition (Brzostek et al. 2015; Soudzilovskaia et al. 2015). The differences in FF properties can be attributed to contrasting leaf and root chemical traits between AMF and EMF trees, which influence the palatability and litter decomposability of their litter and the accumulation of soil organic matter (Phillips et al. 2013). Compared with AMF trees, EMF trees produce more hyphal necromass and lower‐quality litter, promoting slower decomposition and thicker FFs (Vesterdal et al. 2013). Moreover, EMF can suppress decomposition under nutrient‐poor conditions by competing with saprotrophic fungi, a process known as the Gadgil effect (Gadgil and Gadgil 1971; Mayer, Matthews, et al. 2023; Figure 3e). The underlying mechanisms and generality of this effect remain uncertain (Fernandez et al. 2020; Choreño‐Parra and Treseder 2024). In coniferous forests, EMF hyphae are considered important drivers of FF accumulation (Högberg et al. 2020). At the same time, EMF secrete extracellular enzymes involved in organic matter degradation (Meier et al. 2015; Carteron et al. 2020), enabling host trees to acquire organic and inorganic N from Of and Oh FF horizons (McGuire et al. 2013; Khokon et al. 2021, 2023). Consequently, the importance of EMFs in plant nutrition from FFs may increase from mull to moder and mor humus forms (Figure 3e). Since AMF have limited capacity to produce hydrolytic extracellular enzymes, they are more closely associated with mineral soil, and their supply of nutrients to host trees depends largely on nutrient mobilization by saprotrophic microbes (Talbot et al. 2008; Lin et al. 2017) and mineral nutrient pools. The occurrence of mycorrhizal fungi is further influenced by host root biomass. In cold moist, acidic, and nutrient‐poor forests, fine roots are concentrated in thicker FF layers (Leuschner, Wiens, et al. 2006; Finér et al. 2007; Meier et al. 2018), resulting in high EMF abundance here (Lilleskov et al. 2019).

Finally, knowledge on FF layer‐specific composition and functionality of microbial communities is still rare as well as knowledge on the influence of FF microbiota on the microbiome of the mineral soil beneath. Observations at local (Wang et al. 2021) as well as latitudinal scales (Shi et al. 2014) have indicated that effects are likely. The degree of accumulation of FF mass may be the result of trophic interactions. However, quantitative general knowledge about these interrelationships along the FF layers is missing, and studies comparing FF versus mineral soil indicate different overall interactions across different trophic groups (Mundra et al. 2021).

## 4 Forest Changes Cause Forest Floor Changes

The high turnover of FFs and their close linkage to environmental conditions (Figure 2) make FFs particularly sensitive to ongoing forest changes.

### 4.1 Nitrogen and Sulfur Deposition and Acidification

Past input of S and N widely impacted biochemical cycles in natural ecosystems (de Vries et al. 2014). Especially between 1960 and 1990, S and N inputs induced pH declines in forest soils, and high N input also caused eutrophication of forest ecosystems (Schöpp et al. 2003). It is reasonable to assume that the deposition dynamics up to the 1990s are still reflected in the properties of FFs. Acidification leads to losses of K, Mg, and Ca, and increasing release of Al ions (Al^3+^) as well as heavy metals into the soil solution. The availability of base cations (Desie et al. 2020; Villalba‐Ayala et al. 2025), Al (Leuschner, Meier, and Hertel 2006), and Mn (Roth et al. 2022; Spohn and Stendal 2023) under acidification will likely remain crucial factors in controlling FF turnover. Acidification also suppresses microbial activity and impairs soil macrofauna, thereby reducing litter decomposition (Shen et al. 2021; Růžek et al. 2021). Over the last three decades, S and N emissions have declined across Europe, and several studies indicate that reduced deposition rates have already altered the soil C cycle (Oulehle et al. 2011). Accordingly, Grüneberg et al. (2019) suggested that the FF mass loss observed in Germany is related to increased decomposition in response to rising soil pH.

Deposition of N deposition is still high in many temperate forests. Due to effective retention, N accumulates in soils and can trigger ecological responses even at low doses if sustained over time (Phoenix et al. 2012). Yet, the potential impact of increased N deposition on FF thickness remains controversial. Observed negative correlations between N availability and FF thickness suggest that N deposition may mitigate N limitation and cause decreasing FF thickness (Forstner et al. 2019). In contrast, other studies showed that high N concentrations can impair decomposition. Reasons might be inhibited lignin degradation (Berg and Matzner 1997; Bonner et al. 2019) or decreasing need to mine organic matter for N (Fernandez et al. 2020). Also, interlinkages between N inputs and P cycling might increase FF thickness: Several studies indicated that high N inputs to forest ecosystems increase the risk of nutritional imbalances between N and P (Peñuelas et al. 2012; Jonard et al. 2015; Krüger et al. 2020), likely mediated by mycorrhizal changes (Bidartondo et al. 2026). The P supply of trees is closely linked to FF properties and turnover (Vesterdal and Raulund‐Rasmussen 1998; Lang et al. 2017). The P concentration of leaf litter shapes the microbial decomposer community (Bergkemper et al. 2016), which may contain more than 20% of total P in FFs (Bauhus and Khanna 1999). Nitrogen‐induced P deficiency in forests may cause accumulation of FF mass due to depletion of litter P upon intense resorption of P from senescing tissues by trees (Sohrt et al. 2018), and thus, rendering litter less decomposable. In their recent review, Kuyper et al. (2023) conclude that decreased decomposition is the dominant impact of N deposition on C cycling. In summary, the current continuously high N deposition load induces lower turnover and higher mass of FFs even under overall decreasing acid deposition.

Climate change effects are becoming more prominent around the globe, with climatic variations (e.g., high temperatures, drought, extreme weather events) and associated biological disturbances (e.g., shifts in plant, microbial and animal communities) significantly impacting the structure and functioning of forest ecosystems (Helm et al. 2017; Seidl et al. 2020; Mayer et al. 2024). The FF is particularly sensitive to climatic disturbances that alter its defining processes, such as litter decomposition (Callesen et al. 2003; Krishna and Mohan 2017). Short‐term effects of increasing temperatures at sufficient moisture induce increased microbial activity and C mineralization rates; long‐term effects cause increased C losses, particularly in FFs, due to absent or only little protection by association with minerals. Several studies already demonstrated the vulnerability of FFs to climate warming, reporting decreases in C stocks of FFs (Wellbrock et al. 2017; Prietzel et al. 2016; Prietzel, Falk, et al. 2020) and increased losses of DOC (Borken et al. 2011). In support, elevation gradient studies show faster FF turnover and decreasing FF mass thickness with increasing air temperatures (Oulehle et al. 2011; Labaz et al. 2014; Hagedorn et al. 2019; Moreno‐Duborgel et al. 2025). Accordingly, a recent study provides evidence for high sensitivity of microarthropods in the FF to climatic changes (Chen et al. 2025). Longer periods without precipitation might reduce litter decomposition, and thus, increase FF mass (Bottner et al. 2000; Guidi et al. 2023). Based on a survey across France, Ponge et al. (2011) concluded that climate warming might have opposing effects on FF thickness, depending on water availability: a decrease under sufficient water supply and an increase under dry conditions due to reduced decomposition (Bottner et al. 2000; Guidi et al. 2023). Peak mineralization after re‐wetting (Brunn et al. 2023) does not (over)compensate the decreased decomposition during dry periods. Adding even more complexity, Baldrian et al. (2013) emphasized that consecutive warmer winters may elevate microbial activities during an otherwise dormant season, which has been confirmed by recent observations of increased ecosystem C release in winter (Liu et al. 2020; Sanders‐DeMott et al. 2020). In summary, climate change‐related drivers of FF changes are (1) increasing temperature, which will accelerate soil microbial and faunal activity at sufficient moisture, thereby decreasing FF mass, (2) decreasing soil moisture, which will result in increasing FF mass unless sufficiently strong decomposition peaks after re‐wetting, and (3) a combination of both with unpredictable consequences. Thus, the resulting changes in FFs are highly uncertain.

Natural forest disturbances such as wildfires, windthrow, droughts, and insect infestations have increased in recent decades, and ongoing climate change is predicted to foster this trend (Seidl et al. 2017; Haberstroh et al. 2022; Patacca et al. 2022). Natural disturbances can have strong and long‐lasting effects on the FF by reducing litter input and promoting losses via erosion or changes in microclimatic conditions (Thom et al. 2023). Meta‐analyzes of the effects of natural disturbances suggest a general decline in FF stocks following stand disturbance (Zhang et al. 2015; Nave et al. 2022; Mayer et al. 2024). Wildfires can cause rapid losses by direct combustion of FF material and may trigger reduced net primary productivity (Pellegrini et al. 2018; Mack et al. 2021). Wildfires also induce the formation of pyrogenic organic matter characterized by high biogeochemical stability (Schmidt et al. 2011; Pellegrini et al. 2022). Thus, fire‐prone forests may have particularly large C stocks in the mineral soil despite concurrent FF losses during single fire events (Eckmeier et al. 2010). Large reductions in FF stocks have been reported from mountain forests damaged by windthrow, possibly due to increased surface erosion and temperature‐related acceleration of organic matter decomposition (Gerber et al. 2002; Christophel et al. 2015; Mayer et al. 2024). Soil mixing via uprooting of trees may result in the burial of FF beneath mineral soil layers (Stutz and Lang 2023). Insect infestations have smaller impacts on FFs following stand damage than wildfire and windthrow (Mayer et al. 2024). This may be due to the input of frass debris (e.g., litter, faeces) into the FF, off‐setting losses following infestations (Le Mellec and Michalzik 2008; Yang and Gratton 2014). The duration and magnitude of FF losses in response to natural disturbances appear to be most pronounced when FF layers are thick, while thin FF layers appear more resilient to stand damage (Mayer et al. 2024). In summary, literature indicates a decrease in FF mass through forest disturbance and emphasizes that the vulnerability of FFs to environmental changes is context specific and might increase with FF mass.

Tree species selection, stand structure and density control, and removal of biomass by harvesting represent the most relevant forest management practices. Tree species composition is one major FF control (see also Section 3; Guckland et al. 2009; Prietzel and Bachmann 2012; Vesterdal et al. 2013), which may even result in tree species‐specific humus forms under similar climatic conditions (Prietzel and Bachmann 2012; Bayranvand et al. 2017). The impact of tree species on FF accumulation can be traced back to the quantity and quality of leaf and root litter (Binkley and Giardina 1998), root exudates (Meier et al. 2017), and to the impact mediated via effects on soil microorganisms and fauna (Hobbie et al. 2006). Even single trees can locally affect FF and soil processes if they differ enough from their surroundings (Stutz and Lang 2023). Previously, intensive management of temperate forests promoted a high share of coniferous over broadleaved species. Conifer litter has a lower decomposition rate because of having a higher lignin:N ratio than deciduous leaf litter (Prescott et al. 2000; Leuschner and Ellenberg 2017). The high contents of tannins and other protein‐precipitating polyphenols in conifer litter may further hamper decomposition processes (Stempfhuber et al. 2017). Accordingly, higher FF mass has been observed with increasing contribution of conifers (Wilcke et al. 2024; Moreno‐Duborgel et al. 2025). Mixing broadleaved species into previously conifer‐dominated stands in many Central European forests has been shown to accelerate the litter decomposition rate (Achilles et al. 2021), causing shifts in humus forms from moder/mor to mull/moder (Labaz et al. 2014). Also, the spatial variability in FF masses and humus forms at stand level was found to be higher in mixed than pure stands (Aubert et al. 2006).

Besides the slower decomposition of conifer litter, also the microclimatic conditions in dense, mostly younger and even‐aged, pure (conifer) stands may inhibit microbial processes, thus slowing down decomposition and, hence, fostering FF accumulation (Albers 2004; Berger and Berger 2012, 2014). The structure and openness of the canopy have a strong influence on moisture availability and temperature at the ground, both of which affect rates of decomposition and nutrient mineralization. Uneven‐aged and mixed stands, characterized by higher structural complexity, often have lower diurnal temperature ranges and vapor pressure deficits than even‐aged and pure stands (Ehbrecht et al. 2017, 2019; Schnabel et al. 2025). For European beech, the litter concentrations of nutrients, such as N, Mg, and K decrease with stand age, causing lower litter decomposition rates (Trap et al. 2013).

Silvicultural measures, such as thinning (Vesterdal et al. 1995) or harvesting of mature trees, can affect the decomposition of foliar litter and nutrient mineralization. Clear cutting or gap harvesting causes marked increases in soil nitrate concentration due to reduced microbial assimilation in response to reduced C input (Bauhus 1996; von Wilpert et al. 2000; Prescott 2002; Prescott et al. 2003) and, perhaps to a greater magnitude, reduced nitrate uptake by trees (Ritter and Vesterdal 2006; Schleppi et al. 2017; Ward and Bradford 2025). To summarize, forest management practices feedback to FF drivers, mainly forest microclimate, litter quality and quantity, and soil pH. How management impact is modified by climate change or by other environmental changes remains largely unresolved.

## 5 Forest Floors: Ecosystem Hubs at Risk

Our synthesis of the literature prompts us to call for a paradigm shift. Instead of viewing the FF as an ecological anomaly, particularly in the case of intensive accumulation of organic matter, it should be recognized as an integral part of forests, resulting from interactive adaptations to environmental conditions. As an interface between the atmosphere, biosphere, and pedosphere (Studer et al. 2026), the FF is unique in terms of its position, size, extent, and vulnerability. We emphasize that the FF reflects the ecosystem's response to site nutrient status, soil acidity, temperature, and precipitation, mediated by biotic interactions that evolved over millions of years (Ponge 2013). The long‐term adaptation of plants and soil organisms to specific combinations of environmental factors determines not only the habitat quality of FFs, but also energy and element fluxes within forests. Depending on FF turnover, crucial processes of the C, nutrient, and water cycles take place in the FF (Figure 3a–e): with slow FF turnover, energy and element cycling within the FF is tight and supports forest nutrition, water retention, and microbial transformation of litter. With fast FF turnover, mineralization and fragmentation of aboveground litter occur in the FF and elements are translocated into the mineral soil, the groundwater, or back into the atmosphere.

**FIGURE 4:**
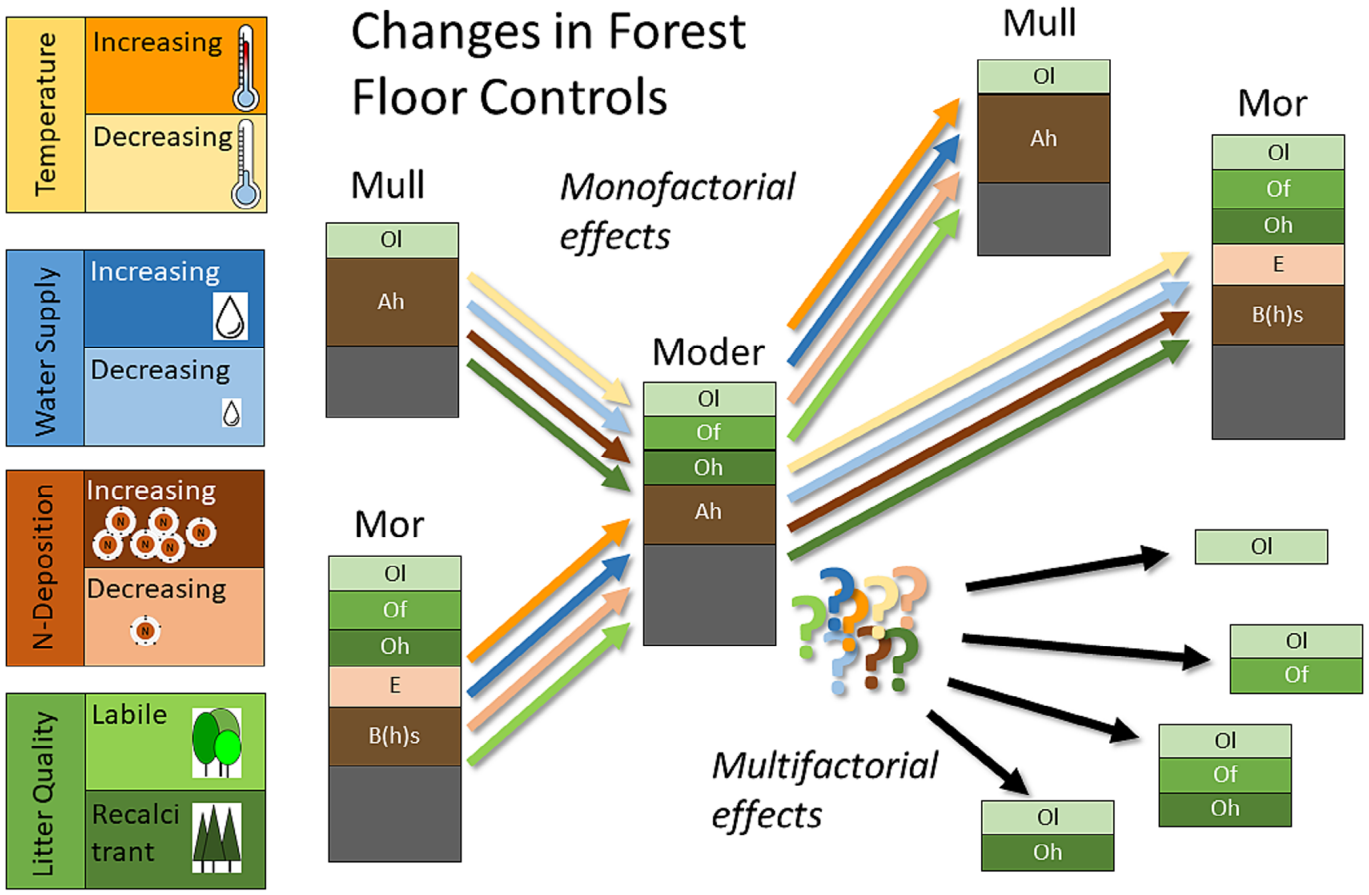
Forest floor controls, on the left, might change due to shifts either in climate conditions (e.g., temperature or water supply), environmental conditions (e.g., N deposition), or changes in forest management practice (e.g., tree‐species‐specific litter quality). Horizon labelling according the German classification system (Wachendorf et al. 2023). Proposed impacts of monofactorial controls (colored arrows) on the forest floor are covered in model assumptions (Zanella, Berg, et al. 2018; Zanella et al. 2022; Wachendorf et al. 2023), for (the more likely) multifactorial dependencies of such changes (black arrows), the expected outcome on forest floor properties remains unclear.

The strong interconnectedness of FF processes to the mineral soil and the aboveground ecosystem compartments justifies defining the FF as the “central ecosystem hub.” As outlined below, the tight interconnectedness of processes and multifactorial dependencies (Figure 4) explain the vulnerability of FFs to environmental change, which calls for new avenues for research and management.

### 5.1 Interconnectedness

The soil biota of the FF translates different human‐induced changes into changing the cycling of nutrients, C, and water, thereby shifting their availability, uptake, and storage. More so, processes in the FF control its development via strong feedbacks to organisms, water, nutrient, and C fluxes (e.g., litter input drives microbial release of nutrients that feeds back to plant growth and litter input; Stutz 2023). Exploring the efficiency of the manifold interactions, mechanisms, and organisms, as well as how they link to mineral soil and aboveground ecosystem functioning is challenging. Nevertheless, understanding such interconnectedness is crucial for assessing FF vulnerability against forest change and offers great potential for the development of forest health indicators. A promising approach is the linking of morphological properties (Section 2) to specific ecosystem processes.

### 5.2 Multifactorial Dependencies

This term highlights that the numerous factors regulating FF processes and FF morphology are not strictly additive regarding their impact, meaning that the response of FFs to specific factors is co‐regulated by the characteristics of other factors. Trade‐offs between ecosystem adaptation to different factors are particularly likely when novel combinations of factors rapidly emerge and create conditions the ecosystem has never experienced before and for which no suitable adaptation strategies have been developed. This happens to be the case for the human impacts on global element and energy cycles. We conclude that such novel combinations of controlling factors pose a major threat to the functionality of the FF–mineral soil continuum.

Human‐induced N deposition illustrates the *decoupling of factors*. Over decades, N deposition has affected the interactions of factors controlling FF properties. In forest ecosystems unaffected by N deposition, nitrate concentrations in soil solution are controlled by nitrification rates. Net nitrification increases with pH and temperature, and in the past mainly soils showing higher pH and warmer climate featured high nitrate concentrations. Since N deposition peaked in the 70's and 80's, nitrate concentrations in soils affected by N deposition have become less controlled by soil temperature, pH, or other plant nutrients, resulting in fundamental biogeochemical changes (de Vries and Du 2024) and tree nutrition imbalances (Peñuelas et al. 2020).

Recent changes in temperature and soil acidification provide an impressive example of *disproportional changes* causing novel combinations of environmental factors. At a global scale, the occurrence of acid soils is linked to high precipitation and low temperatures (Slessarev et al. 2016). This is because high leaching rates support continuous soil base cation depletion, which supports decreases in soil pH. Yet, natural soil acidification in humid regions is a slow process slowly reversible. Fast temperature increases accompanied with decreasing precipitation could cause pronounced disequilibria between modern climatic conditions and soil properties, thereby changing the interplay of factors controlling FFs and ecosystems. Processes, organisms, and forests benefiting from temperature‐limited decomposition of litter and the associated accumulation of FF material would be especially impaired by climate warming and result in highly vulnerable FFs.

### 5.3 Open Questions

Assessing FF vulnerability and developing appropriate management strategies require answers to several research questions:

- How does the interconnectedness of controlling factors, key organisms, and key processes shape the structural and functional characteristics of FFs?
- Which combinations of environmental factors are hazardous and constrain the provision of certain FF ecosystem services?
- How does the high sensitivity of the FF to natural and human factors feedback to the functioning of mineral topsoil horizons and the overall functioning of forest ecosystems?

An essential starting point for addressing these questions is agreement on a unified global terminology for describing FFs. This would enable the comparison between different regions and would help extract generic information on the vulnerability of FFs around the globe. Furthermore, FF research strictly requires interdisciplinary approaches for exploring the interactions among key organisms in relation to FF characteristics. Promising opportunities to target the above‐mentioned research questions include: (1) observational studies at sites with specific combinations of controlling factors, (2) long‐term monitoring, including retrospective analyzes of archived FF samples, (3) experiments with different types of labelled litter to trace the fate of C and nutrients in combination with defined access of soil organism groups to FFs, and (4) experimental manipulations of FF properties.

## Author Contributions

**Friederike Lang:** conceptualization, investigation, funding acquisition, writing – original draft, writing – review and editing, methodology, project administration. **Jörg Prietzel:** investigation, methodology, funding acquisition, writing – original draft, writing – review and editing, conceptualization. **Lars Vesterdal:** methodology, investigation, writing – original draft, writing – review and editing, conceptualization. **Jürgen Bauhus:** investigation, writing – original draft, writing – review and editing, funding acquisition. **Maï Bergmann:** investigation, writing – original draft, writing – review and editing. **Sebastian Bibinger:** investigation, writing – original draft, writing – review and editing. **Klaus Kaiser:** conceptualization, investigation, writing – review and editing, methodology. **Martin I. Bidartondo:** investigation, writing – review and editing. **Simon Haberstroh:** investigation, writing – original draft, writing – review and editing. **Frank Hagedorn:** conceptualization, investigation, funding acquisition, writing – original draft, methodology, writing – review and editing. **Jonas Hahn:** investigation, writing – original draft, writing – review and editing. **Peter Hartmann:** investigation, writing – original draft, writing – review and editing. **Mathias Mayer:** investigation, methodology, writing – review and editing, conceptualization, writing – original draft. **Jingxuan Olivia Chen:** investigation, writing – original draft, writing – review and editing. **Philipp de Jong:** investigation, writing – original draft, writing – review and editing. **Ina C. Meier:** investigation, funding acquisition, writing – original draft, writing – review and editing. **Trung Hieu Doan:** investigation, writing – original draft, writing – review and editing. **Heike Puhlmann:** investigation, funding acquisition, writing – original draft, writing – review and editing. **Anis Mahmud Khokon:** investigation, writing – original draft, writing – review and editing. **Stefanie Schulz:** conceptualization, investigation, funding acquisition, writing – original draft, writing – review and editing, methodology. **Martin Kohler:** investigation, writing – original draft, writing – review and editing. **Laura M. Suz:** investigation, writing – original draft, writing – review and editing. **Heinke Paulsen:** investigation, writing – original draft, writing – review and editing. **Helmer Schack‐Kirchner:** investigation, funding acquisition, writing – original draft, writing – review and editing. **Michael Schloter:** investigation, writing – original draft, writing – review and editing. **Lexie Schilling:** investigation, writing – original draft, writing – review and editing. **Patrick Schleppi:** investigation, writing – original draft, writing – review and editing. **Jörg Niederberger:** conceptualization, investigation, writing – original draft, methodology, writing – review and editing, visualization, project administration. **Kenton P. Stutz:** investigation, writing – original draft, writing – review and editing. **Gabriela Villalba:** investigation, writing – original draft, writing – review and editing. **Richard Neumann:** investigation, writing – original draft, writing – review and editing. **Markus Weiler:** investigation, funding acquisition, writing – original draft, writing – review and editing. **Melissa Wannenmacher:** investigation, writing – original draft, writing – review and editing. **Christiane Werner:** investigation, funding acquisition, writing – original draft, writing – review and editing. **Kai Schwärzel:** investigation, funding acquisition, writing – original draft, writing – review and editing. **Nicole Wellbrock:** investigation, writing – original draft, writing – review and editing. **Stefan Scheu:** investigation, funding acquisition, writing – original draft, writing – review and editing.

## Funding

This work was supported by Deutsche Forschungsgemeinschaft (Grant LA 1398/19‐1), (457330547).

## Conflicts of Interest

The authors declare no conflicts of interest.

## Supporting information


**Table S1:** Definitions and horizon assignments for organic horizons by selected soil or humus form classification systems.

**Table S2:** Examples of selected Humus form‐ and soil‐systematics or classifications, describing a humus form (see Tables S1 and S3); focusing of the three at all classification system present humus forms Mull, Moder and Mor; with typical horizon sequence* and specific requirements that must be met to qualify as the specific humus form.

**Table S3:** Selection of accessible* international and national soil classification systems, defining organic horizons and or humus forms; humus form classification or taxonomy systems.

## Data Availability

Data sharing not applicable to this article as no datasets were generated or analyzed during the current study.
