# Supplementary material for: Temperate Forest Floors: Ecosystem Hubs in Transition?
Source: Glob Chang Biol. 2026 Aug 18;32(8):e71033. doi: 10.1111/gcb.71033 (PMC13482849; doi:10.1111/gcb.71033)
Supplement: Supplementary file 1 [file GCB-32-e71033-s001.pdf]

Suppplenentary Table S1: Definitions and horizon assignments for organic horizons by selected soil or humus forrm classification systems

<table>
<tr><td colspan="6" rowspan="3">Definitions of Organic horizons</td><td>1)
WRB 4th ed. (12/2022)</td><td>2)
US Soil taxonomy (Keys to soil taxonomy, 13th ed. 2022)</td><td>3)
The Canadian system of soil classification</td><td colspan="2">4)
KA6 (Germany)</td><td colspan="2">5)
Zanella et al.</td></tr>
<tr><td colspan="7">distinction to mineral soil</td></tr>
<tr><td>≤ 90% recognizalbe dead plant tissue; ≥ 20% organic carbon [m/m]</td><td>≥ 12% organic carbon [m/m]</td><td>≥ 17% organic carbon [m/m]</td><td colspan="2">≥ 15% organic carbon [m/m]</td><td colspan="2">≥ 20% organic carbon [m/m]</td></tr>
<tr><td>orginic fine material [v/v]</td><td>recognizable plant tissue [v/v]</td><td>State of decomposition</td><td colspan="3">Structure</td><td>Organic horizon</td><td>Organic horizon</td><td>Organic horizon</td><td>Organic horizon</td><td>Sub-type</td><td>Organic horizon</td><td>Sub-type</td></tr>
<tr><td>0-10</td><td>100-90</td><td>no or hardly</td><td colspan="3">loose, not glued or densly packed</td><td colspan="2">litter layer</td><td rowspan="3">L</td><td>Ol</td><td>Ol</td><td>OL</td><td>nOL / vOL</td></tr>
<tr><td>10-30</td><td>90-70</td><td rowspan="3">initial, minor desintegration of plant tissue, origin easy recognicable</td><td colspan="3" rowspan="5">stacked, glued, bendable, cross-linked, felted</td><td rowspan="2">Oi</td><td rowspan="4">Oi</td><td rowspan="5">Of</td><td>Olf</td><td rowspan="5">OF</td><td rowspan="5">zoOF/ nozOF</td></tr>
<tr><td>30-35</td><td>70-65</td><td rowspan="4">Ohf</td></tr>
<tr><td rowspan="2">35-60</td><td rowspan="2">65-40</td><td rowspan="4">Oe</td><td rowspan="4">F</td></tr>
<tr><td rowspan="2">intermediate, largley desintegration of plant tissue, origin of parts of plant tissue still recognicable</td></tr>
<tr><td>60-70</td><td>40-30</td><td rowspan="2">Oe</td></tr>
<tr><td>70-85</td><td>30-15</td><td rowspan="2">far advanced, major desintegration of plant tissue, origin of organic substance not or hardly recognizable</td><td rowspan="2">powdery, crumbly, brittle</td><td rowspan="2">diffuse break-able</td><td rowspan="2">sharp break-able</td><td rowspan="2">Oh</td><td rowspan="2">Oih/Obh/ Oxh/Okh/ Ovh/Osh/ Odh/...</td><td rowspan="2">OH</td><td rowspan="2">zoOH/ szoOH/ nzoOH</td></tr>
<tr><td>85-100</td><td>15-0</td><td>Oa</td><td>Oa</td><td>H</td></tr>
</table>

[1] WRB: World Reference Base for Soil Resources. International soil classification system for naming soils and creating legends for soil maps. 4th edition 2022; [2] US Soil Taxonomy - Keys to Soil Taxonomy, 13th edition. USDA Natural Resources, 2022; [3] The Canadian System of Soil Classification, 3rd edition, 1998; [4] KA6: Bodenkundliche Kartieranleitung KA6, German soil classifiction system 6th edition, 2024; [5] proposed European Humus Form Reference Base, Zanella et al. 2018d;
