# Supplementary material for: Temperate Forest Floors: Ecosystem Hubs in Transition?
Source: Glob Chang Biol. 2026 Aug 18;32(8):e71033. doi: 10.1111/gcb.71033 (PMC13482849; doi:10.1111/gcb.71033)
Supplement: Supplementary file 2 [file GCB-32-e71033-s002.docx]

| Classification/  Current version | Focus | Litter included | O horizon, requirements |  | **Humus form** | |
| --- | --- | --- | --- | --- | --- | --- |
|  |  |  |  |  | typical horizon sequence | |
|  |  |  |  |  | specific requirements | |
|  |  |  |  | **Mull** | **Moder** | **Mor (Rohumus)** |
| Germany  Bodenkundliche Kartieranleitung 2024,  6. Auflage | Soil | yes | OC ≥ 15 % (w/w) | (Ol)-(Of)-Ah/Ax/Au | Ol-Of-Obh-Ah (Ee) | Ol-Of-Osh/Okh-  Ah-Ee/Ee-Ah |
|  |  |  |  | - no Oh, - Ah present | non | Sharp transition O to A or E horizon |
| Zanella et al., 2018 | Humus form | yes | OC ≥ 20 % (w/w) | (OL)-maA | OL-zoOF-zoOH-miA | OL-nozOF-(szoOH)-AE/EA |
|  |  |  |  | - no OH - ma A | - OH - no nozOF - no maA | - no maA, meA - nozOF, - pH_H2O_ < 4.5 - A absent or structure massive/single grain - sharp transition O to A/E horizon |
| Austria  Österreichische Bodensystematik  2011 | Soil | yes | OM ≥ 35 % (w/w) | L-(Fzo)-Ah | L-F-H-Ah | L-F-H-Ah/Ae |
|  |  |  |  | non | L < F and H | - L, F and H present - L+F+H>5cm, - Sharp transition O to A horizon |
| France  Référentiel pédologique  2008 | Soil | yes | OC ≥ 17-20 % (w/w) | (Ol)-A | OL-OFzo-OHzo-A | OL-OFnoz-  (OHnoz)-E/(Eh) |
|  |  |  |  | - maA - Sharp transition O to A horizon | - miA - diffuse transition O to A horizon | - Sharp transition O to A horizon |
| Poland  Roczniki Gleboznawcze  2011 | Soil | yes |  | Ol/A | Ol-Ofh-A | Ol-Of-Oh-A |
|  |  |  |  | non | non | non |

**Sup. Table S2:** Examples of selected Humus form- and soil-systematics or classifications, describing a humus form (see supp. Tab. S1 and supp. Tab. S3); focusing of the three at all classification system present humus forms Mull, Moder and Mor; with typical horizon sequence* and specific requirements that must be met to qualify as the specific humus form.

| Classification/  Current version | Focus | Litter included | O horizon, requirements |  | **Humus form** | |
| --- | --- | --- | --- | --- | --- | --- |
|  |  |  |  |  | typical horizon sequence | |
|  |  |  |  |  | specific requirements | |
|  |  |  |  | **Mull** | **Moder** | **Mor (Rohumus)** |
| Swiss:  Klassifikation der Böden der Schweiz  2010, 3. Auflage | Soil | yes | OM > 30% (M/M) | Ol-(Of)-Ah | Ol-Of-(Oh)-Ah/(EAh) | Ol-Of-Oh-(EAh)/AE |
|  |  |  |  | non | non | non |
| Canada:  The Canadian system of soil classification, 1998, 3^rd^ edition, respectively Green et al (1993) | Soil | yes | OC > 17% (w/w) | (L)-(Fz)-Ah | L-Fz-(Hz)-AH | L-Fm-Hh/Hr-(Ah) |
|  |  |  |  |  | - Fz, diagnostic horizon | - Fm, diagnostic horizon - Sharp transition to A horizon |

***) Horizon in brackets**: horizon is possible or appears only periodically; **Ol, OL, L**: horizon consisting of organic material in an initial state of decomposition; **Of, OF, F**: horizon consisting of organic material in a more advance state of decomposition; **Oh, OH, H**: consisting of organic material in far advanced state of decomposition, organic fine matter is the principal component; **A-horizon:** mineral topsoil horizon**; E-horizon:** mineral soil horizon, characterized by eluvial processes; **maA:** biomacrostructured A-horizon; **meA:** biomesostructured A-horizon; **miA**: biomicrostructured A-horizon; **noz**: non zoogenic; **zo**: zoogenic; **szo**: slightly zoogenic; **OM**: organic matter; **OC**: organic carbon; **Fz**: F horizon zoogenic origin; **Fm**: F horizon mycogenic origin; **Hz**: H horizon, zoogenic origin; **Hh**: H-Horizon dominated by OFM; **Hr**: Hh horizon with high portion of plant residues.
