# Supplementary material for: Temperate Forest Floors: Ecosystem Hubs in Transition?
Source: Glob Chang Biol. 2026 Aug 18;32(8):e71033. doi: 10.1111/gcb.71033 (PMC13482849; doi:10.1111/gcb.71033)
Supplement: Supplementary file 3 [file GCB-32-e71033-s003.docx]

**Sup. Table S3:** Selection of accessible*^)^ international and national soil classification systems, defining organic horizons and or humus forms; humus form classification or taxonomy systems.

| **Region or system** | **Classification** | **Version** | **Definitions** | |
| --- | --- | --- | --- | --- |
|  |  |  | Organic Horizons | Humus Form |
| **International /**  **Organizational** | World Reference Base for Soil Resources (WRB) | 2022,  4^th^ edition | Yes | No |
|  | FAO Guidelines for Soil Description | 2006,  4^th^ edition | Yes | No |
|  | ICP Forest Manual Part X: Sampling and Analysis of Soil | 2020-1 | Yes | No |
| **National** |  |  |  |  |
| USA | USDA soil taxonomy | 2012,  Version 3.0 | Yes | No |
| Canada | The Canadian System of Soil Classification | 1998,  3^rd^ edition | Yes | Yes^§)^ |
| New Zealand | New Zealand Soil Classification | 2010  3^rd^ edition | Yes | No |
| Australia | The Australian Soil Classification ^%^ | 3^rd^ edition  Online version (14 June 2023 | Yes | No |
| Germany | Bodenkundliche Kartieranleitung | 2024,  6. Auflage | Yes | Yes |
| Austria | Österreichische Bodensystematik | 2011 | Yes | Yes |
| France | Référentiel pédologique | 2008 | Yes | Yes |
| Suisse | Klassifikation der Böden der Schweiz | 2010,  3. Auflage | Yes | Yes |
| Poland | Systematyka Gleb Polski | 2011 | Yes | Yes |
| Russia | Russian Soil Classification system | 2001 | Yes ^$)^ | Yes ^$)^ |
| Brazil | Sistema Brasileiro de  Classificação de Solos | 2019.  6th edition | Yes ^#)^ | No |
| **Humus form classification** | German Soil Science Society, Working group humus forms | 2024 | Yes | Yes |
|  | Zanella et al. | 2018 | Yes | Yes |
|  | Green et al. | 1993 | yes | yes |
|  | Klinka et al. | 1981 | yes | yes |

*) accessible in terms of either an English version available or one of the authors could read the original version.

§) the Canadian Soil classification define organic horizons and use humus forms to distinguish between soil types, for humus form definition the authors reference Green et al (1993).

%) the Australian soil classification us the USDA Soil taxonomy for describing organic horizons

$) the Russian classification describes a various number of different organic horizons and humus forms.

#) the Brazilian System knows an organic horizon with various different possible properties, leading to different organic soils, but no different organic horizons or humus forms.

**References**: all links below: last access 10.06.2026

Organizational:

IUSS Working Group WRB. (2022). World Reference Base for Soil Resources. International soil classification system for naming soils and creating legends for soil maps. 4th edition. International Union of Soil Sciences (IUSS), Vienna, Austria. [https://wrb.isric.org/]

FAO (Food and Agriculture Organization of The United Nations) (2006). Guidelines for soil description, Fourth edition, FAO, Rome. [https://www.fao.org/4/a0541e/a0541e.pdf]

Cools N, De Vos B, 2020: Part X: Sampling and Analysis of Soil. Version 2020-1. In: UNECE ICP Forests Programme Co-ordinating Centre (ed.): Manual on methods and criteria for harmonized sampling, assessment, monitoring and analysis of the effects of air pollution on forests. Thünen Institute of Forest Ecosystems, Eberswalde, Germany, 29 p + Annex [http://www.icp-forests.org/manual.htm]

Soil Survey Staff (1999). Soil Taxonomy - A Basic System of Soil Classification for Making and Interpreting Soil Surveys, 2^nd^ edition, USDA Natural Resources Conservation Service, Washington. [https://www.nrcs.usda.gov/sites/default/files/2022-06/Soil%20Taxonomy.pdf]

Soil Survey Staff (2022). Keys to Soil Taxonomy, 13th edition. USDA Natural Resources Conservation Service, Washington. [https://www.nrcs.usda.gov/sites/default/files/2022-09/Keys-to-Soil-Taxonomy.pdf]

National:

NRC/CNCR (National Research Council of Canada), (1998). The Canadian system of soil classification 3rd edition, National Research Council of Canada, Ottawa. [https://sis.agr.gc.ca/cansis/publications/manuals/1998-cssc-ed3/index.html]

Hewitt, A. E. (2010). New Zealand Soil Classification, 3^rd^ edition, Landcare Research New Zealand Ltd, Manaaki Whenua Press, Lincoln N.Z. [https://soils.landcareresearch.co.nz/topics/soil-classification/nzsc]

Isbell, R. F., National Committee on Soil and Terrain (2021). The Australian Soil Classification, 3^rd^ edition, CSIRO Publishing, Melbourne. [https://www.soilscienceaustralia.org.au/asc/]

AG Boden (2024). Bodenkundliche Kartieranleitung, 6. Aufl., Band 2 Geländeaufnahme und Systematik, Bundesanstalt für Geowissenschaften und Rohstoffe, Hannover. [https://www.bgr.bund.de/SharedDocs/Meldungen/DE/Boden/2025-02-03_KA6-erschienen.html]

Nestroy, O., Aust, G., Blum, W. E. H, Englisch, M., Hager, H., Herzberger, E., Kilian, W., Nelhiebel, P., Ortner, G., Pecina, E., Pehamberger, A., Schneider, W., Wagner, J. (2011). Österreichische Bodensystematik 2000 in der revidierten Fassung von 2011, Österreichische Bodenkundliche Gesellschaft, Wien.

AFES (Association française pour l’étude du sol) (2008). Référentiel pédologique, Edition Quæ, Versailles. [https://www.afes.fr/wp-content/uploads/2023/10/Referentiel_Pedologique_2008.pdf]

BGS (Bodenkundliche Gesellschaft der Schweiz) (2010). Klassifikation der Böden der Schweiz, 3. Auflage, BGS, Luzern. [https://www.soil.ch/de/publikationen/klassifikation]

Systematyka gleb Polski (2019). Polskie Towarzystwo Gleboznawcze, Komisja Genezy Klasyfikacji i Kartografii Gleb. Wydawnictwo Uniwersytetu Przyrodniczego we Wrocławiu, Polskie Towarzystwo Gleboznawcze, Wrocław–Warszawa. [https://ptgleb.pl/en/materials/polish-soil-classifications/]

Shishov, L. L., Tonkonogov, V. D., Lebedeva, I. I., Gerasimova, M. I. (2001). Russian Soil Classification Sytem, Dokuchaev Soil Science Institute, Moskow,

Embrapa (2018). Sistema Brasileiro de Classificação de Solos, 5ª edição revista e ampliada, Emprapa, Brasília, DF. [https://www.embrapa.br/en/tema-solos-brasileiros/solos-do-brasil]

Humus form:

Wachendorf, C., Frank, T., Broll, G., Beylich, A., Milbert, G. (2023). A Concept for a Consolidated Humus Form Description—An Updated Version of German Humus Form Systematics, Int. J. Plant Biol., 14, 658-686. https://doi.org/10.3390/ijpb14030050

Zanella, A., Ponge, J. F., Jabiol, B., Sartori, G., Kolb, E., Gobat, J.-M., Le Bayon, R.-C., Aubert, M., De Waal, R, et al. (2018d). Humusica 1, article 5: Terrestrial humus systems and forms – Keys of classification of humus system forms. Applied Soil Ecology 122, 75-86. http://dx.doi.org/10.1016/j.apsoil.2017.06.012

Green, R. L., Trowbridge, R. N., Klinka, K. (1993). Towards a taxonomic classification of humus forms. Society of American Foresters, Forest Science Monograph 29. https://doi.org/10.1093/forestscience/39.s1.a0001

Klinka, K., Green, R. N., Trowbridge, R. L., & Lowe, L. E. (1981). *Taxonomic Classification of Humus Forms in Ecosystems of British Columbia* (No. 8; Landscape Management Report, p. 54). Ministery of Forest. [https://open.library.ubc.ca/soa/cIRcle/collections/facultyresearchandpublications/52383/items/1.0107268]
